# Obesity and risk for hypertension and diabetes among Kenyan adults: Results from a national survey

**DOI:** 10.1097/MD.0000000000027484

**Published:** 2021-10-08

**Authors:** Tecla M. Temu, Paul Macharia, James Mtui, Martin Mwangi, Paul W. Ngungi, Celestine Wanjalla, Gerald S. Bloomfield, Carey Farquhar, Loise Nyanjau, Gladwell K. Gathecha, Joseph Kibachio

**Affiliations:** a Department of Global Health, University of Washington, Seattle, WA; b Institute of Tropical Diseases, University of Nairobi, Nairobi, Kenya; c Consulting in Health Informatics, Kenya; d Saint George University, University Center, Grenada, West Indies; e Ministry of Health, Nairobi, Kenya; f Kenya National Bureau of Statistics, Nairobi Kenya”; g Department of Medicine, Vanderbilt University, Nashville, TN; h Duke Global Health Institute, Duke Clinical Research Institute, Department of Medicine, Duke University, Durham, NC; i Department of Medicine and Epidemiology, University of Washington, Seattle, WA; j World Health Organization, Pretoria, South Africa.

**Keywords:** Africa, diabetes, hypertension, Kenya, obesity

## Abstract

Despite the anticipated growth in the global burden of obesity especially in low-income countries, limited data exist on the contribution of obesity to cardiometabolic diseases in Africa.

We examined population-based samples of Kenyan adults who participated in the 2015 national chronic disease risk factor surveillance survey. Weight and height were measured, and body mass index (BMI) was calculated and used as a measure for general obesity. Waist circumference (WC), a clinical measure of central obesity was also measured. Logistic regression was used to assess the association between obesity with hypertension, diabetes, and dyslipidemia risk.

Of the 4276 participants, the median (IQR) age was 36 (27–47) years, 41% were men. One-third (37%) of the participants were centrally obese, whereas 10% were generally obese. The odds for overweight and general obesity were highest among females, adults >40 years, and those in the highest wealth quartile. Central and general obesity, assessed by WC and BMI, were associated with hypertension and dyslipidemia but not diabetes for both sexes. Compared with adults of normal weight, individuals with a BMI of ≥30 kg/m^2^ had an odds ratio of 2.39 (95% confidence interval [CI], 1.82–3.12) for hypertension and 2.24 (95% CI, 1.70–2.96) for dyslipidemia.

Obesity prevalence is high in Kenya and is associated with hypertension and dyslipidemia but not diabetes. Our findings indicate an urgent need to develop public health interventions to address obesity and prevent the development of comorbid conditions.

## 1 Introduction

Cardiovascular diseases (CVD) such as stroke and coronary heart diseases account for 33% of all global deaths, with over two-thirds of those deaths occurring in low- and middle-income countries.^[1]^ Data on causes of death in the sub-Saharan Africa (SSA) countries including Kenya are limited but recent estimates show that CVD may account for 11% of deaths, an increase of 81% between 1990 and 2013.^[2]^ Obesity, a condition related to excess fat adiposity, is associated with an increased risk for dyslipidemia, diabetes, and hypertension which are independent and major risk factors for CVD.^[3–7]^ Obesity prevalence is increasing globally, and present estimates show that 1 of 5 people living in urban settings in Africa are either overweight or obese.^[8,9]^ At the same time, the prevalence of hypertension, diabetes, and dyslipidemia has also been on the rise in African countries, consistent with the upward trend of the rates of obesity.^[9–12]^

Body mass index (BMI) and waist circumference (WC) are simple, cheap, and commonly used clinical measures for general and central adiposity respectively.^[13,14]^ Previous studies in the United States have reported a weaker association between BMI with hypertension and diabetes among African Americans compared to their white counterparts. In some minority populations, a J-shaped relationship and, in some cases, no association has also been reported between measures of abdominal adiposity with diabetes and hypertension suggesting that factors linking obesity and health condition may be ethnic and gender specific.^[4,13,15,16]^ Despite this, very few studies have examined the utility of different measures of adiposity in predicting risk for diabetes, dyslipidemia, and hypertension in Africans particularly for those residing in the rural regions.^[9]^ Most of the earlier studies from SSA were conducted among individuals recruited from health facilities and failed to evaluate the gender specific differences of obesity on health conditions. Most importantly, they all focused on a single measure of obesity and almost all were restricted to the association between obesity and hypertension alone.^[5,17]^ As a result, it is still unclear which metric of obesity (BMI or WC) best correlates with the cardiovascular health of Africans. Clarifying the relationship of different measures of obesity with obesity-related conditions is critical in planning intervention strategies and policy decisions to control obesity and prevent CVD, cancers, and other health-related complications in Africa.

Using data from a large nationally representative sample, we evaluated patterns of obesity in the urban and rural population in Kenya, and examine the association of hypertension, diabetes, and dyslipidemia with different measures of obesity by sex.

## 2 Methods

### 2.1 Data source

The 2015 Kenya STEPs survey was the first national cross-sectional household survey on non-communicable diseases (NCD) risk factors conducted in Kenya. Information about this survey have been previously described in detail.^[18]^ Briefly, this survey used a multistage stratified sampling method to allow national estimates by age, sex, and residence (urban and rural). The survey used the fifth National Sample Surveys and Evaluation Programme (NASSEP V) master sample frame that was developed by the Kenya National Bureau of Statistics (KNBS). The frame was developed using the Enumeration Areas (EAs) generated from the 2009 Kenya Population and Housing Census to form 5360 clusters split into four equal sub-samples. A total of 6000 households were sampled targeting one individual randomly selected from all eligible household members. A total of 4725 adults gave consent and 4500 eligible adults between 18–70 years old were successfully interviewed among the households, yielding a response rate of 75%. To produce unbiased estimates, sampling weights were calculated as the inverse or reciprocal of all the selection probabilities at all the stages mentioned above. The weighting derived from the processes involved in the creation of sampling frame and post-stratification procedures were performed to compensate for unequal probabilities of selection, to adjust for non-response, and to ensure that the results are consistent with population data according to age-sex categories. For this analysis, we further excluded 224 observations with incomplete data on WC, weight, and height measurements. Thus, a total of 4276 individuals were included in the final analysis.

### 2.2 Ethical approval

The study protocol was reviewed and approved by the Kenya Medical Research Institute's Ethics Review Committee. All eligible participants gave an informed written consent.

### 2.3 Key variables

Body weight and height were measured to the nearest 0.1 kg and 0.1 cm, respectively, by using standardized equipment (SECA 877) and procedures.^[18]^ WC was measured to the nearest 0.1 cm at the level of the iliac crest.^[18]^ Body mass index (BMI) was calculated from weight and height measurements. We used cut-off points as proposed by the World Health Organization where a BMI of 25 to 29.9 kg/m^2^ is termed overweight and a BMI of ≥0 kg/m^2^ is considered general obesity.^[19]^ Central obesity was defined using the International Diabetes Federation (IDF) as WC of ≥80 cm (women) and ≥94 cm (men) of African ancestry.^[20]^ Three blood pressure measurements were obtained at 2-minute intervals with the subject in a seated position by using a validated battery powered OMRON automated blood pressure machine. The average of the 3 readings was used to define the systolic blood pressure (BP) and diastolic BP levels. Hypertension was defined when the mean showed systolic BP ≥140 mmHg or diastolic BP ≥90 mmHg, or if the participant reported being on antihypertensives per Kenyan guidelines.^[21]^ Blood samples were obtained after a minimum 12-hour fast for biochemical measurements (blood glucose and cholesterol). Diabetes mellitus was defined as fasting blood glucose (FBG) ≥7 mmol/L (126 mg/dL) or use of diabetes medications. Dyslipidemia was defined as total cholesterol (TC) ≥5.2 mmol/L (200 mg/dL) or high-density cholesterol (HDL) <1.03 mmol/L (men, 40 mg/dL) or <1.30 mmol/L (women, 50 mg/dL). Individuals who had >1 of the following CVD risk factors were considered to have multiple comorbidities: hypertension; diabetes; and dyslipidemia.

### 2.4 Potential confounding variables

Data on other covariates such as age, place of residence (urban or rural), health behaviors (ie, smoking, alcohol, physical activity), marital status, education, diet information, and household socioeconomic status were obtained directly from the study participants using items from the World Health Organization STEP wise approach to chronic disease risk factor surveillance. Socioeconomic status was measured using a household asset and amenities index commonly used in Demographic and Health Surveys in low and middle-income countries.^[22]^ Standardized weight scores were generated using principal components analysis and ranked to generate wealth quintiles which were recoded into five quintiles with the lowest representing poorest households to the highest quintile representing the wealthiest households. Vigorous physical activity for work was defined as carrying or lifting heavy loads, digging or construction work for 10 minutes continuously. Physical activity for travel was defined as walking or using a bicycle for at least 10 minutes to get to and from places. Leisure time physical activity was defined as engaging in sports, fitness, or recreation for at least 10 minutes continuously. Fruit and vegetable consumption was recorded in terms of number of servings per day and number of days per week. Adequate fruit and vegetable intake was defined as consuming ≥5 servings of fruits or vegetables per day. Present smokers were defined as present users of smoked tobacco. present alcohol intake was defined as any reported alcohol consumption in the past year.

### 2.5 Statistical analysis

Descriptive statistics for all variables stratified by sex were analyzed using chi-square test for categorical variables and using *t* test or its nonparametric counterpart for continuous variables that were not normally distributed. To evaluate the association between overweight/obesity and sociodemographic factors, logistic regression analysis was applied to estimate the odds ratio (OR) of overweight/obesity through levels of various explanatory factors. The adjusted OR was presented with a 95% confidence interval (CI). We also used separate logistic regression models to examine the relationship between obesity measures (BMI and WC classifications) with hypertension, diabetes, dyslipidemia, and a presence of ≥2 comorbidities (hypertension, diabetes, dyslipidemia) for each sex separately. The normal weight BMI and normal WC were used as the reference category. The regression models were adjusted for the potential confounding variables including age, marital status, health behaviors (smoking, alcohol, physical activity), residence (urban and rural), education level, and socioeconomic status. These variables were chosen based on their associations with hypertension, diabetes, and dyslipidemia. Analyses were performed using STATA version 13 (San Antonio, TX). The geospatial analyses were performed using QGIS version 3.14.0 “Pi”. QGIS is a free and open-source cross-platform desktop geographic information system application that supports analysis of geospatial data.

## 3 Results

**Figure 1:**
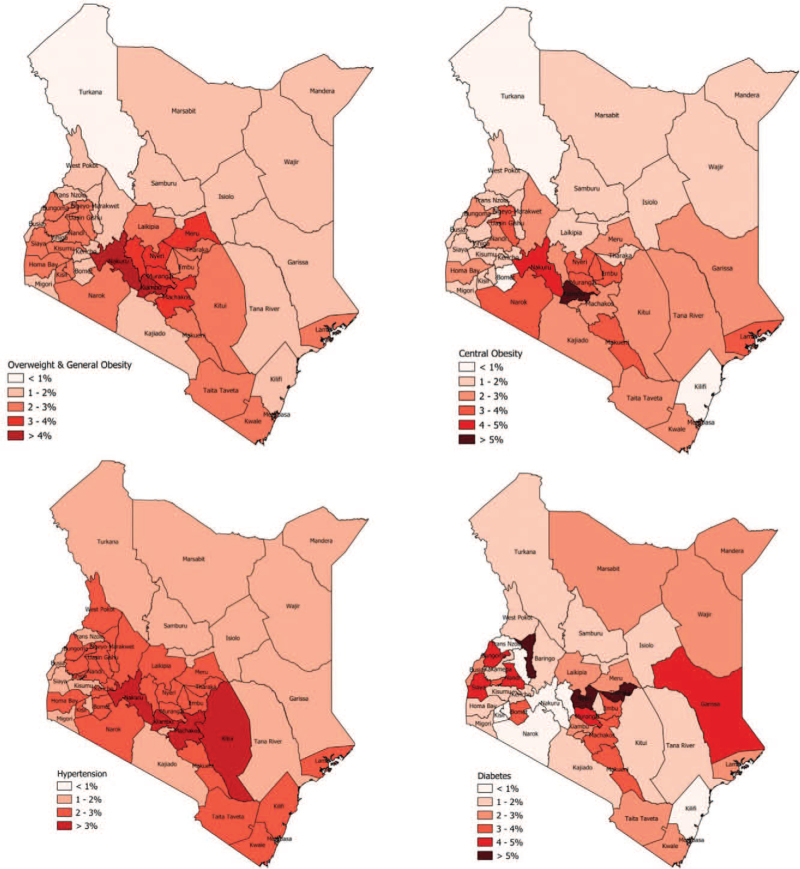
Estimated adult obesity, hypertension, and diabetes prevalence by County in Kenya.

Table 1 shows the characteristics of the 4,276 participants included in this analysis by sex. The median (IQR) age of the population was 36 (27–47) years and 41 were male. Majority of the participants were <40 years of age. Overall, 10% of all individuals were generally obese and 37% were centrally obese. Females had significantly higher mean BMI, WC, fasting blood glucose, and total cholesterol than males (*P* < .001; for all). Men were more likely to smoke and drink alcohol; they were also likely to report physical activity that met WHO recommendations when compared to women (Table 1). Diabetes rates were low and more common among females than males (2.9% vs 1.6%, *P* < .001); rates of hypertension (28.9% vs 29.9%, *P* = .49) and dyslipidemia (7.5% vs 8.8%, *P* = .13) were similar between the 2 sexes (Table 1). Thirty percent of the participants had at least one of these cardiovascular risk factors, and 5.2% had multiple comorbidities. Figure 1 reports estimated adult obesity, hypertension, and diabetes prevalence by county in Kenya.

**Table 1:** Sociodemographic and other selected characteristics of adults participants of the 2015 National Chronic Disease Risk Factor Survey, by Sex.

	Total	Female	Male	*P*
	N = 4276	N = 2520	N = 1756	
Outcomes
Age, y	38.1 ± 13.3	38.1 ± 13.6	38.1 ± 13.1	.97
Obesity-related variables				
WC, cm	79.6 ± 13.7	80.0 ± 14.4	79.0 ± 12.7	<.001
BMI, kg/m^2^	23.4 ± 5.2	24.4 ± 5.6	22.2 ± 4.3	.02
Overweight	887 (21)	633 (25)	254 (14)	<.001
General Obesity	446 (10)	360 (14)	86 (5)	<.001
Centrally Obesity	1561 (37)	1258 (50)	303 (17)	<.001
Fasting glucose, mg/dL	84 ± 25	86 ± 25	82 ± 26	<.001
SBP, mmHg	127 ± 20	126 ± 21	130 ± 19	<.001
DBP, mmHg	83 ± 12	83 ± 12	82 ± 12	.03
Total cholesterol, mg/dL	143 ± 39	147 ± 40	136 ± 37	<.001
Elevated BP	1253 (29.4)	728 (28.9)	525 (29.9)	.49
Diabetes	103 (2.4)	75 (2.9)	28 (1.6)	.001
Dyslipidemia	343 (8.0)	189 (7.5)	154 (8.8)	.13
Demographics				
Marital status				<.001
Never married	760 (18.8)	344 (14.5)	416 (24.9)	
Divorced /separated	410 (10.1)	356 (14.9)	54 (3.2)	
Married/cohabitating	2876 (71.1)	1681 (70.6)	1195 (71.8)	
Education				<.01
None	697 (21.6)	537 (28.6)	160 (11.9)	
Primary school or below	943 (29.3)	557 (29.7)	386 (28.7)	
Secondary school	1094 (33.9)	572 (30.5)	522 (38.8)	
College and above	487 (15.3)	210 (11.3)	277 (20.6)	
Residence				<.001
Rural	2195 (51.4)	1359 (54.1)	836 (47.8)	
Urban	2073 (48.5)	1155 (45.9)	918 (52.3)	
Wealth				<.001
Poorest	862 (20.2)	558 (22.2)	304 (17.3)	
Second	846 (19.8)	497 (19.8)	349 (19.9)	
Third	861 (20.1)	523 (20.8)	338 (19.3)	
Fourth	850 (19.9)	470 (18.7)	380 (21.6)	
Wealthiest	849 (19.9)	466 (18.5)	383 (21.8)	
Present smokers	381 (8.9)	21 (0.8)	360 (20.5)	<.001
Present alcohol drinkers	1427 (33.4)	447 (17.8)	980 (55.9)	<.001
Moderate/vigorous activity	1922 (45.0)	1047 (41.6)	875 (49.9)	<.001
Healthy diet	2454 (57.6)	1448 (57.7)	1006 (57.5)	.90

Data reported as mean ± standard deviation, percentage, or median (interquartile range [IQR]). BMI = body mass index, BP = blood pressure, DBP = diastolic BP, SBP = systolic BP, SD = standard deviation, WC = waist circumference.

Factors associated with overweight and general obesity by sex are shown in Supplementary Table 1 (Supplemental Digital Content, on factors associated with overweight and general obesity). After adjustment of demographic and lifestyle factors, overweight and general obesity was commonly reported among women compared to men (odds ratio [OR], 2.70, 95% CI; 2.34–3.12); individuals in the wealthiest quintiles compared to those in the poorest quintiles (OR 1.58, 95 CI% 1.50–2.66); and married people compared to the never married (OR 1.37, 95% CI 1.21–1.56). Older individuals (>40 years) were also 59% more likely to be overweight/obese than the younger (<40 years) individuals (OR 1.59, 95% CI 1.40–1.81).

The results of the logistic regression in which obesity measures (BMI or WC) were used to predict obesity-related comorbidities are shown in Table 2. Individuals with central (OR 2.02; 95% CI 1.67–2.44) and general obesity (OR 2.39, 95% CI 1.82–3.12) were twice or more likely to have elevated blood pressure. They were also at higher odds of having dyslipidemia and multiple comorbidities (*P* < .001; Table 2). Obesity phenotype by BMI was a stronger predictor for hypertension, dyslipidemia, and presence of multiple comorbidities than the WC phenotype (Table 2). We did not observe any significant association between general or central obesity with diabetes. Details of these associations by sex are reported in Supplementary Table 2a and 2b (Supplemental Digital Content, which demonstrate the association between overweight and obesity with hypertension, dyslipidemia, and diabetes by sex).

**Table 2:** Adjusted odds ratios for selected metabolic conditions by body mass index category and waist circumference.

	Hypertension^∗^	Diabetes^∗^	Dyslipidemia^∗^	≥2 Comorbidities
	AOR (95% CI)	AOR (95% CI)	AOR (95% CI)	AOR (95% CI)
Body mass index, kg/m^2^				
Normal: 18.5–<25	1 (Ref)	1 (Ref)	1 (Ref)	1 (Ref)
Underweight: <18.5	0.68 (0.50–0.91)	1.18 (0.78–1.77)	0.91 (0.72–1.15)	0.48 (0.22–1.07)
Overweight: 25 to <30	1.64 (1.33–2.03)	1.29 (0.93–1.79)	1.97 (1.62–2.42)	2.68 (1.72–4.18)
Obese: BMI ≥30	2.39 (1.82–3.12)	1.42 (0.95–2.14)	2.24 (1.70–2.96)	4.89 (2.94–8.14)
Waist Circumference, cm				
Normal WC	1 (Ref)	1 (Ref)	1 (Ref)	1 (Ref)
Central obese	2.02 (1.67–2.44)	1.32 (0.99–1.77)	1.77 (1.48–2.10)	4.02 (2.67–6.08)

yAOR = adjusted odds ratio, BMI = body mass index, CI = confidence interval, WC = waist circumference.

∗ Adjusted for age, socioeconomic status, marital status, education level, residence (rural vs urban), health behaviors (physical activity, smoking, and alcohol intake).

## 4 Discussion

In this nationally representative sample of Kenyan adults, we found high rates of general, and central obesity; these phenotypes were significantly associated with being female, older age >40 years, highly educated, married, and wealth. Our study extends literature by further identifying sex-specific contribution of central and general obesity toward diabetes, dyslipidemia, and hypertension at the population level. We utilized multiple measures of obesity which has never been previously explored before in sub-Saharan Africa region (SSA). We found that generally and centrally obese adults were twice as likely to have hypertension and dyslipidemia and 4 times likely to have multiple co-morbidities than their normal-weight and normal WC counterparts regardless of sex. We however found no relations between obesity and being diabetic.

The reported high rates of obesity among women and older age individuals in our study is consistent with findings globally and in other SSA countries.^[10,11,23]^ Surprisingly, we did not observe statistically significant association between obesity and urban residency after adjustment for including, marital status, education, and wealth level. This is in contrast with previous studies in SSA and other developed countries that demonstrated an increase likelihood of being overweight/obese among those residing in urban areas.^[5,11,23]^ The urbanization of the rural areas in Kenya and the expansion of the peri-urban may have blunt the effect of urban lifestyle including poor nutrition and sedentary lifestyle. Previous studies in Kenya have reported a high prevalence of obesity among Kenyan slum residents who do not necessarily fit into the classic definition of rural-urban.^[24]^ It is important to also point out that the previous studies in the region lacked data on socioeconomic status and therefore were not able to adjust for it. Our study results highlight an urgent need for intervention to prevent and treat overweight and obesity on the population wide basis. On an individual level, structured programs that emphasize on lifestyle changes including education, reduced fat, and physical activity can produce long-term weight loss.

Previous studies of metabolic risks of obesity in SSA have typically used body mass index (BMI) to assess for general obesity even though various regional fat distributions may have different metabolic implications.^[5,10,17]^ As a result, very little information exists regarding the contribution of central obesity on metabolic related diseases. Likewise, almost most of these studies did not assess the relationship between obesity with dyslipidemia or diabetes.^[5,10,17]^ Here in, we report for the first time that Kenyan adults with central and general obesity regardless of sex had double the risk for hypertension and dyslipidemia compared to normal weight and those without central obesity. The observed risks for hypertension due to obesity noted in this investigation are consistent with data from the South Africa,^[5]^ Malawi,^[11]^ and Nigeria^[17]^ that also reported a strong association of general obesity with hypertension. Our findings are also consistent with the recent data from the Cardiovascular H3Africa Innovation Resource study conducted in 13 African countries, which found that both generalized and central obesity is. associated with hypertension.^[10]^ We did not have any data on obesity association with dyslipidemia from the region for comparison. However, our data are consistent with data from similar studies performed in the high-income countries.^[4,25]^ Our results suggest that both measures of obesity are cheap and an effective way to assess adults at risk for cardiometabolic complications. Future studies that include other morbidities such as hospitalizations, stroke, coronary heart disease and cancers are needed to investigate outcomes and to assess the long-term economic and health consequences of obesity.

Obesity has been frequently associated with diabetes in the developed world. Not much research has been done in the SSA region to clarify the influence of central or general obesity on diabetes. A cross-sectional study across four low- and middle-income countries reported an increased risk for diabetes among the overweight and generally obese South African adults compared to the normal weight adults.^[5]^ In our analysis, we did not find any significant association between obesity measures and diabetes in both sexes. The discrepancy in our findings compared to these studies could be due to several factors. One, the pathogenesis and risk factors of diabetes in low-income countries may differ slightly from those in much high-income countries including South Africa.^[26]^ Secondly, the data from the South Africa study was derived from tertiary health institutions in urban areas and may not equally apply for individuals in the rural areas. Lastly, the very low rates of diabetes possibly due to younger age of our representative sample may have made these findings statistically insignificant and therefore our study need to be interpreted with caution. Additional research using similar sampling methods are needed in Africa to confirm these findings.

Our study had several strengths. It is the first study to assess the relationship of obesity with diabetes and elevated cholesterol in SSA using national wide data and standardized methods among individuals. Since we had fasting measures of glucose and cholesterol, we were able to accurately assess for diabetes and dyslipidemia status. Limitations of our study included the cross-sectional design, therefore the directionality of the relationship between obesity and metabolic diseases can’t be established. We also relied on single BP measurement while guidelines recommend obtaining the mean using multiple BP measurements obtained during ≥2 visits for the diagnosis of hypertension. Similarly, we relied on self-reported measures for sociodemographic and lifestyle risk factors hence we cannot preclude the possibility of bias. We also lacked data on early life adverse factors such as birth weight and prematurity, and the women reproductive history.

In conclusion, the burden of obesity-related morbidity and mortality has not been well documented in SSA. The long-term health and economic consequences are likely to be significant considering the existing poor health systems, lack of qualified health professionals, and the increase rates of obesity in this region. With increased urbanization, physicians are likely to encounter an increasing number of individuals with obesity-related health conditions across all ages and sex. Although there have been efforts in Kenya and other African countries to identify and control hypertension, dyslipidemia, and diabetes initiatives to promote a balance diet, physical activity, and weight control should also become a public health priority.

## Acknowledgments

The authors thank everyone who took part in the design and implementation of the main survey. This funding for the main survey was provided by World bank, WHO, AstraZeneca, and MOH/CDC. Data collection was done by the Ministry of Health, WHO and Kenya National Bureau of statistics, Kenya Medical Research Institute (KEMRI), and African Institute for Health and Development (AIHD).

## Author contributions

TT, CF, GG, JK, JM, MM, PN, EW, PM contributed to the conception and design of the study, the supervision, data acquisition, analysis and interpretation, and the critical revision of the manuscript. TT, PM, and CW accessed and verified the underlying data. GG, JK, LN, PN, MM, JM, CW, EW, CF, PM, and GSB contributed to the data analysis, the data interpretation, the manuscript drafting, and the critical revision of the manuscript.

**Conceptualization:** Tecla Temu, Paul Macharia, James Mtui, Martin Mwangi, Paul W. Ngungi, Celestine Wanjalla, Gerald S. Bloomfield, Carey Farquhar, Loise Nyanjau, Gladwell K. Gathecha, Joseph Kibachio.

**Data curation:** Paul W. Ngungi, Celestine Wanjalla, Loise Nyanjau, Gladwell K. Gathecha, Joseph Kibachio.

**Formal analysis:** Tecla Temu, Paul Macharia, James Mtui, Paul W. Ngungi, Celestine Wanjalla, Gerald S. Bloomfield, Carey Farquhar, Gladwell K. Gathecha.

**Funding acquisition:** Loise Nyanjau, Joseph Kibachio.

**Methodology:** Tecla Temu, Martin Mwangi, Paul W. Ngungi, Celestine Wanjalla, Gerald S. Bloomfield, Loise Nyanjau, Gladwell K. Gathecha, Joseph Kibachio.

**Project administration:** Martin Mwangi, Paul W. Ngungi, Loise Nyanjau, Joseph Kibachio.

**Writing – original draft:** Tecla Temu, Paul Macharia, James Mtui, Martin Mwangi, Paul W. Ngungi, Celestine Wanjalla, Gerald S. Bloomfield, Carey Farquhar, Loise Nyanjau, Gladwell K. Gathecha, Joseph Kibachio.

**Writing – review & editing:** Tecla Temu, Paul Macharia, James Mtui, Martin Mwangi, Paul W. Ngungi, Celestine Wanjalla, Gerald S. Bloomfield, Carey Farquhar, Loise Nyanjau, Gladwell K. Gathecha, Joseph Kibachio.

## Supplementary Material

Supplemental Digital Content

## Supplementary Material

Supplemental Digital Content

## Supplementary Material

Supplemental Digital Content
