# Supplementary material for: Obesity and risk for hypertension and diabetes among Kenyan adults: Results from a national survey
Source: Medicine (Baltimore). 2021 Oct 8;100(40):e27484. doi: 10.1097/MD.0000000000027484 (PMC8500651; doi:10.1097/MD.0000000000027484)
Supplement: Supplemental Digital Content [file medi-100-e27484-s001.docx]

**Supplementary Table 1:** Adjusted odds ratios for overweight/obesity in relation to social-demographic factors among Kenya adults by sex.

|  | **Female** | | **Male** | |
| --- | --- | --- | --- | --- |
|  | **Age adjusted** | **Full Adjustment** | **Age adjusted** | **Full Adjustment** |
|  | **OR (95% CI)** | **OR (95% CI)** | **OR (95% CI)** | **OR (95% CI)** |
| Age, years |  |  |  |  |
| < 18 | 1 | 1 | 1 | 1 |
| 40-49 | 1·67 (1·34-2·06) | 1·89 (1·41- 2·53) | 2·34 (1·75-3·14) | 2·64 (1·81-3·87) |
| 50+ | 1·36 (1·12-1·66) | 2·85 ( 2·07-3·90) | 1·73 (1·28-2·32) | 2·08 (1·38-3·12) |
| Marital status |  |  |  |  |
| Never married | 1 | 1 |  |  |
| Married/cohabitating | 1·14 (0·89-1·46) | 2·07 (1·50-2·71) | 2·18 (1·60-2·98) | 2·73 (1·79-4·15) |
| Divorced/separated | 0·78 (0·56-1·10) | 1·53 (0·99-2·36) | 1·52 (1·09-2·09) | 1·08 (0·32-3·56) |
| Education |  |  |  |  |
| None | 1 | 1 | 1 | 1 |
| Less than secondary | 3·98 (2·99-5·31) | 2·03 (1·43-2·88) | 2·29 (1·25-4·18) | 1·76 (0·86-3·59) |
| Secondary | 4·19 (3·12-5·63) | 1·74 (1·20-2·52) | 2·84 (1·58-5·10) | 1·78 (0·89-3·56) |
| More than secondary | 7·10 (4·90-10·29) | 2·23 (1·41-3·52) | 7·64 (4·21-13·86) | 3·60 (1·76-7·34) |
| Residence |  |  |  |  |
| Rural | 1 | 1 | 1 | 1 |
| Urban | 1·93 (1·64-2·28) | 0·97 (0·76-1·23) | 2·71(2·09-3·51) | 1·28 (0·89-1·85) |
| Wealth |  |  |  |  |
| Poorest | 1 | 1 | 1 | 1 |
| Second | 2·33 (1·74-3·13) | 2·16 (1·44-3·26) | 1·75 (0·10- 3·20) | 1·98 (0·93-4·20) |
| Third | 3·69 (2·77-4·90) | 3·23 (2·17-4·82) | 3·09 (1·75-5·43) | 2·56 (1·25-5·26) |
| Fourth | 5·95 (4·44-7·96) | 4·91 (3·22-7·50) | 4·41 (2·55-7·62) | 2·94 (1·44-5·98) |
| Wealthiest | 8·60 (6·38-11·60) | 7·37 (4·64-1·69) | 14·73 (8·65-25·07) | 7·78 (3·78-16·03) |
| Alcohol Intake |  |  |  |  |
| Never | 1 | 1 | 1 | 1 |
| Current | 0·89 (0·72-1·11) | 0·84 (0·63-1·11) | 0·86 (0·68-1·10) | 0·81 (0·60-1·10) |
| Smoking status |  |  |  |  |
| Never | 1 | 1 | 1 | 1 |
| Current smoker | 0·35 (0·11-1·03) | 0·18 (0·02-1·11) | 0·36 (0·25-0·53) | 0·40 (0·24-0·65) |
| Physical activity |  |  |  |  |
| Sedentary | 1 | 1 | 1 | 1 |
| Active | 0·71 (0·60-0·84) | 0·98 (0·77-1·23) | 0·40 (0·32-0·52) | 0·71 (0·51-0·98) |
| Fruit and vegetable intake |  |  |  |  |
| Met recommendation | 1 | 1 | 1 | 1 |
| Did not meet recommendations | 1·37 (1·16-1·630 | 1·19 (0·96-1·49) | 1·22 (0·95-1·56) | 0·93 (0·69-1·26) |
| Other abbreviations as in Table 1. The models are adjusted for age, socio-economic status, marital status, education level, residence, physical activity. | | | | |
