# Supplementary material for: Obesity and risk for hypertension and diabetes among Kenyan adults: Results from a national survey
Source: Medicine (Baltimore). 2021 Oct 8;100(40):e27484. doi: 10.1097/MD.0000000000027484 (PMC8500651; doi:10.1097/MD.0000000000027484)
Supplement: Supplemental Digital Content [file medi-100-e27484-s002.docx]

**Supplementary Table 2a:** Adjusted odds ratios for selected metabolic disorders prevalence by body mass index category and sex

|  | **Hypertension ^a^** | **Diabetes ^a^** | **Dyslipidemia ^a^** | **≥2 Comorbidities** |
| --- | --- | --- | --- | --- |
|  | AOR (95%CI) | AOR (95%CI) | AOR (95%CI) | AOR (95%CI) |
| **Female** |  |  |  |  |
| Normal: 18·5 to <25 | 1 (Ref) | 1 (Ref) | 1 (Ref) | 1 (Ref) |
| Underweight: <18·5 | 0·60 (0·38-0·95) | 1·17 (0·64 - 2·11) | 0·91 (0·66 - 1·46) | 0·59 (0·20 - 1·73) |
| Overweight: 25 to <30 | 1·58 (1·20-2·08) | 1·43 (0·95 - 2·15) | 2·06 (1·59 - 1·89) | 2·90 (1·56 - 5·38) |
| Obese: BMI ≥30 | 2·49 (1·80-3·45) | 1·48 (0·92 - 2·39) | 2·35 (1·68 - 2·19) | 5·77 (2·96 - 11·21) |
|  |  |  |  |  |
| **Male** |  |  |  |  |
| Normal: 18·5 to <25 | 1 (Ref) | 1 (Ref) | 1 (Ref) | 1 (Ref) |
| Underweight: <18·5 | 0·77 (0·52-1·16) | 1·24 (0·71-2·20) | 1·11 (0·65-1·29) | 0·40 (0·11-1·41) |
| Overweight: 25 to <30 | 1·81 (1·31-2·53) | 1·09 (0·62-1·95) | 1·88 (1·35-2·61) | 2·54 (1·31-4·91) |
| Obese: BMI ≥30 | 2·03 (1·23-3·39) | 1·15 (0·49-2·74) | 1·95 (1·15-3·30) | 3·43 (1·39-8·43) |
| BMI, Body mass index; AOR, adjusted odds ratio; CI, confidence interval. Adjusted for age, marital status, wealth, residence (rural vs· urban), physical activity, smoking, and alcohol intake. | | | | |
