# Supplementary material for: Obesity and risk for hypertension and diabetes among Kenyan adults: Results from a national survey
Source: Medicine (Baltimore). 2021 Oct 8;100(40):e27484. doi: 10.1097/MD.0000000000027484 (PMC8500651; doi:10.1097/MD.0000000000027484)
Supplement: Supplemental Digital Content [file medi-100-e27484-s003.docx]

**Supplementary Table 2b:** Adjusted odds ratios for metabolic disorders prevalence by waist circumference Category and sex

|  | **Hypertension ^a^** | **Diabetes ^a^** | **Dyslipidemia ^a^** | **≥2 Comorbidities** |
| --- | --- | --- | --- | --- |
|  | AOR (95%CI) | AOR (95%CI) | AOR (95%CI) | AOR (95%CI) |
| **Female** |  |  |  |  |
| Normal WC | 1 (Ref) | 1 (Ref) | 1 (Ref) | 1 (Ref) |
| Central Obesity | 2·25 (1·77-2·87)*** | 1·41 (0·99-2·03) | 1·74 (1·41-2·15)*** | 4·53 (2·52-8·17)*** |
|  |  |  |  |  |
| **Male** |  |  |  |  |
| Normal WC | 1 (Ref) | 1 (Ref) | 1 (Ref) | 1 (Ref) |
| Central Obesity | 1·72 (1·25-2·35)** | 1·18 (0·69-2·02) | 1·80 (1·31-2·46)*** | 3·59 (1·95-6·62)*** |
| WC, waist circumference; AOR, adjusted odds ratio; CI, confidence interval. ^a^ Adjusted for age, wealth, residence (rural vs. urban), physical activity, marital status, smoking, and alcohol intake.**P<* 0·05, ***P<* 0·01, ****P<* 0·001. | | | | |
